# Spatiotemporal dynamics of the biological interface between cancer and the microenvironment: a fractal anomalous diffusion model with microenvironment plasticity

**DOI:** 10.1186/1742-4682-9-36

**Published:** 2012-08-13

**Authors:** Feng-Chou Tsai, Mei-Chuan Wang, Jeng-Fan Lo, Chih-Ming Chou, Yi-Lu Lin

**Affiliations:** 1 Center for Mathematical Biology; Division of Plastic Surgery, Department of Surgery, Taipei Medical University Hospital, Taipei Medical University, Taipei, Taiwan; 2 Department of Mathematics, Taipei Municipal University of Education, Taipei, Taiwan; 3 Institute of Oral Biology, National Yang-Ming University, Taipei, Taiwan; 4 Department of Biochemistry, Taipei Medical University, Taipei, Taiwan

**Keywords:** Cancer, Anomalous diffusion, Metastasis, Integrins, Probability, Random walk, Pólya constant, Tumor self-seeding

## Abstract

**Background:**

The invasion-metastasis cascade of cancer involves a process of parallel progression. A biological interface (module) in which cells is linked with ECM (extracellular matrix) by CAMs (cell adhesion molecules) has been proposed as a tool for tracing cancer spatiotemporal dynamics.

**Methods:**

A mathematical model was established to simulate cancer cell migration. Human uterine leiomyoma specimens, in vitro cell migration assay, quantitative real-time PCR, western blotting, dynamic viscosity, and an in vivo C57BL6 mouse model were used to verify the predictive findings of our model.

**Results:**

The return to origin probability (RTOP) and its related CAM expression ratio in tumors, so-called "tumor self-seeding", gradually decreased with increased tumor size, and approached the 3D Pólya random walk constant (0.340537) in a periodic structure. The biphasic pattern of cancer cell migration revealed that cancer cells initially grew together and subsequently began spreading. A higher viscosity of fillers applied to the cancer surface was associated with a significantly greater inhibitory effect on cancer migration, in accordance with the Stokes-Einstein equation.

**Conclusion:**

The positional probability and cell-CAM-ECM interface (module) in the fractal framework helped us decipher cancer spatiotemporal dynamics; in addition we modeled the methods of cancer control by manipulating the microenvironment plasticity or inhibiting the CAM expression to the Pólya random walk, Pólya constant.

## Background

Cancer behavior is malignant when the cancer invades neighboring tissues or migrates to survive in the ectopic site (metastasis) [1]. The main kinetic features that distinguish malignant cancer from benign tumors or normal tissues are that cancer cells do not remain in situ in a stable condition, but spread rapidly into neighboring and distant sites through lymphatic or vascular routes. The growth, invasion, and metastases occur concurrently in the microenvironment. An emerging paradigm shift challenges the traditional invasion-metastasis cascade model [2,3] and proposes instead a “parallel progression” cancer model. In this new concept, cancer cells are thought to move by a continuous process of “diffusion” or “random walk”, although their natural retraction-protrusion crawling motion is slower than molecular diffusion. The tracking of single molecules has supported the notion that the sub-diffusion of biological matter in non-homogeneous and anisotropic microenvironments is non-Fickian [4].

The context-dependent responses of biological organisms to their microenvironments, including the ECM (extracellular matrix), generate a continuous sequence of states. The dynamic interplay among internal signaling pathways, surface topology, and external microenvironments renders biological processes nonlinear. EMT (epithelial to mesenchymal transition) and its reverse process MET (mesenchymal to epithelial transition) are known to play a role in cancer progression [5]. The biological cell-CAM (cell adhesion molecule)-ECM interface affects function, and vice versa. In other words, each functional cell is related to a set of corresponding molecular complexes in the specific biological module [6]. For example, CAMs provoke homotypic connections among cells (such as E-cadherins) or heterotypic linkage between cells and ECM (such as integrin *α*_*ν*_) in the chemotactic or haptotactic process of cancer cells [7]. Integrins are heterodimeric, transmembrane glycoproteins comprising an *α* chain and a *β* chain; at least 18 *α* and 8 *β* chains have been identified to date. Integrins represent a key element of the biological landscape or surface topology. Integrins also mediate bidirectional signaling, including the promotion of cell proliferation, adhesion, and resistance to apoptosis. Our previous studies verified that the surface topology of various tissues under surface electron microscopy (SEM) was correlated with intracellular complex signaling and CAM expressions (e.g., integrin *α*_*ν*_) [8,9]. The concept of the “epigenetic landscape” identified by Waddington entails describing perturbed intrinsic phenomena from the perspective of the outermost biological surface [10]. Thus, differences in cell-CAM-ECM modules wired together correspond to different biological states of cancer. We investigated how cancer cells migrate and interact with ECMs in the cell-CAM-ECM interface to generate cancer spatiotemporal dynamics [11].

Biomathematical models use discrete, continuous, or hybrid approaches to depict cancer cell migration behavior, especially the diffusion or random walk mode [12,13]. Our model was based on a modified anomalous diffusion theory of cancer cell movement. In summary, our mathematical model included 2 main points, as follows:

### Fractal space-time scaling of diffusion in probability theory

The use of probability theory by Albert Einstein (1905) to determine Brownian motion provided the first calculation of the Avagadro number [14]. We introduced probability theory to provide probability data on cancer cells in different locations at different times. There are theorems of probability such as the return to origin probability (RTOP), which can be applied to analyze the dynamics of specific cancer cell movements. Moreover, the probabilistic data of cancer cell movements can be normalized to the molecular expressions. Thus, the cell-CAM-ECM interface and its related CAM expressions reflect information on time-series alterations of cancer cell position.

Simple diffusion cannot describe cancer dynamics because real world rarely meets ideal conditions (such as symmetry, isotropic or periodic paths of movement, homogenous microenvironment resistance, or consistent internal movement drive). For example, the clinical diagnosis of skin cancers has for a long time relied on the “AB” acronym, where A refers to asymmetry and B refers to the border (irregular). Uneven cell growth and migration are collectively referred to as morphological instability; this condition is the hallmark of cancer, both clinically and theoretically [15]. A Darwinian clonal selection theory of cancers has verified that the cancerous state represents greater heterogeneity and instability than the normal state [16]. Heterogeneity occurs along various biological or spatiotemporal scales, and induces spatial variance in cancer morphology [17,18].

The assumption of even space-time intervals in simple diffusion is inappropriate. Biological phenomena occur in space-time, the dimensions of which cannot be represented solely by the integers of Euclidean space. Non-linearity can be transformed to a scaling problem by space-time fractal factors and diffusion coefficients of specific directionality. That is, cancer cells migrate by random walk with various degrees of space-time jump or anomalous diffusion [19]. The fractal space-time scaling factor is introduced to quantify the extent of heterogeneity or anisotropy.

### The cell-CAM-ECM interface (module) with a key measurable variable

Because the polarity and diversity of cancer morphological progression cannot be traced easily, our strategy is to monitor one of CAMs as a representative factor of cancer that interacts with the specific elements within the microenvironment through the biological cell-CAM-ECM interface. The expressions of integrins, which are located in areas of surface irregularities, are altered as the surface of the cancer cell changes. Because integrins connect cells at distinctive sites in the ECM, different isoforms of integrins can be used to monitor the changes in the specific cell-CAM-ECM interface that correspond to different points in the progression of cancer. For example, integrin *α*_*ν*_ represents a unique feature in the cell-CAM-ECM interface through binding of fibronectin or vitronectin, which appears as cancer cells attach to the ECM during the invasion of vessels. This concept originates from the module. We selected key measurable variables (such as positional probability and CAM expression) to simplify the analysis.

Previous cancer research has focused largely on gene regulation and signaling, rarely tracing the migration routes or spatiotemporal dynamics in the microenvironment cues. We built a mathematical anomalous diffusion model in the modular and fractal scaling manners. The experiments were also performed to verify our model’s in-silico predictions orderly. The essential data were simplified to a single equation with minimal measurable parameters. Furthermore, the microenvironment plasticity was manipulated to control the cell-CAM-ECM interface (module). Our findings may shed light on novel cancer treatments.

## Main text

The analysis of complex dynamic phenomena such as cancer cell migration through the cell-CAM-ECM interface (module) requires a simple but efficient methodology. Phenomenological models have fewer variables compared with molecular descriptions, enhancing their applicability for experimental data.

Cancer cells migrate in anisotropic and heterogeneous microenvironments. Thus, a simple 3D fractal anomalous diffusion equation is built to describe the dynamics using the Hausdorff derivative. The equation at the cell-CAM-ECM interface $\Gamma \left(t\right)$ gives rise to

(1) $$\frac{\partial p\left(\vec{r},\hat{t}\right)}{\partial \hat{t}}=D\Delta p\left(\vec{r},\hat{t}\right)\text{on}\Gamma \left(t\right)\text{,}$$

where $p\left(\vec{r},\hat{t}\right)$ is the positional probability of cancer cells moving in the fractal space-time frame $\left(\vec{r}=\left({x_{1}}^{\beta_{1}},{x_{2}}^{\beta_{2}},{x_{3}}^{\beta_{3}}\right),\hat{t}=t^{\alpha }\right)$*β* and *α* are fractal space and time parameters respectively; $D=\left(\begin{array}{ccc} {\text{D}}_{11} & 0 & 0\\ 0 & {\text{D}}_{22} & 0\\ 0 & 0 & {\text{D}}_{33} \end{array}\right)$ is a diagonal 3 × 3 diffusion matrix (with *D*_*ii*_ representing diagonal entries), and Δ is the Laplacian operator [20]. The probability density function of cancer provides data on cancer cell locations in the time series.

The fractal time-scaling transform was proposed by Hoffmann and Ord and is referred to as the “internal clock” [21,22]. Assuming the Cauchy problem (Dirac delta function distribution $p\left(\vec{r},0\right)=\delta \left(x_{1}\right)\delta \left(x_{2}\right)\delta \left(x_{3}\right)$), the following solution is obtained [23]:

(2) $$p\left(\vec{r},\hat{t}\right)=\frac{1}{\sqrt{\det\left(D\right)}{\left(4\pi \hat{t}\right)}^{\frac{3}{2}}}e^{-\frac{\vec{r}D^{-1}{\vec{r}}^{T}}{4\hat{t}}}$$

Probability is transformed to the molecular concentration by the normalization process, as follows:

(3) $$p\left(\vec{r},\hat{t}\right)=d\left(\vec{r},\hat{t}\right)/\int d\left(\vec{r},\hat{t}\right)d\vec{r}=\delta \left(\vec{r},\hat{t}\right)=\lambda c\left(\vec{r},\hat{t}\right)\text{,}$$

where $d\left(\vec{r},\hat{t}\right)$ is the number of cancer cells in a specific location and time, $\delta \left(\vec{r},\hat{t}\right)$ is the cancer cell concentration on 3D grids, and $c\left(\vec{r},\hat{t}\right)$ is the molecular expression in which the molecular concentration ratio equals the molecular concentration in the cancer sample compared with the molecular concentration in the normal tissue. The variable *λ* is a normalization factor that is based on the number of CAM subtypes that occupy each cancer cell surface. Thus, the probability and related CAM expressions, such as integrins, can be described in the same manner.

In summary, this model makes the following novel predictions, which were verified by our in silico simulation, in vitro cell, and in vivo mice experiments:

1. Based on probability theory, we introduced the key concept of RTOP, as follows:

(4) $$RTOP\approx p\left(0,\hat{t}\right)=\frac{1}{\sqrt{\det\left(D\right)}{\left(4\pi \hat{t}\right)}^{\frac{3}{2}}}$$

Cancer invasiveness does not cause the patient’s death, but metastasis to the vital organs does. Unsteady tumor cells, whether benign or malignant, may move in several ways (growth, invasion, or metastasis). RTOP denotes that the benign or malignant cells migrate but some either return to the original location, or at least remain in the vicinity of the original location. This demonstrated so-called "tumor self-seeding" phenomenon. We focused on the garrisoned cells, those that remain in the original location. By contrast, healthy host cells remain at the original site and never migrate. RTOP predicts that the CAM concentration ratio $c\left(\vec{r},\hat{t}\right)$ in tumors where a number of migrating cells stay in the vicinity gradually approaches a low level as the tumor grows or as time passes. This is depicted by $\left(c∼p\left(0,t^{\alpha }\right)=\frac{1}{\sqrt{\det\left(D\right)}{\left(4\pi t^{\alpha }\right)}^{\frac{3}{2}}}\right)$. The CAM expressions of tumor surface in the different sampling locations are different because of the different return-to-origin paths and diffusion coefficients. However, the data may be constant if the microenvironment with related diffusion is isotropic and homogeneous. Our finding contradicts the traditional view and the tumor-node-metastasis (TNM) cancer staging system, in which molecular expressions increase as a tumor grows.

Pólya and other researchers have proven that RTOP on a 3D periodic lattice is 0.340537 [24]. Because the muscle has a relatively periodic structure, we use benign smooth muscle tumors to verify whether their CAM expressions are close to the Pólya random walk constant. Specimens of human uterine leiomyoma surface (n = 10) and adjacent normal tissues were obtained from patients undergoing resection at Taipei Medical University Hospital (TMUH). The study protocol was approved by the institutional review board of TMUH (OT-03-08-06). We conducted mRNA and western blot protein analysis to quantify integrin *αν* expressions. Scanning electron microscopy (SEM) was further used to examine the surface of benign tumors.

2. The transition of spatiotemporal probability provides evidence of alterations in the cancer state. Thus, the curve of probability or related cell-CAM-ECM modular expressions can show the spatiotemporal pattern of cancer cell migration.

We cultured three different cell lines (Lewis lung cancer (LLC), human squamous cell carcinoma (SAS) and human pancreatic carcinoma-1 (PANC-1) in the Dulbecco modified Eagle medium (DMEM) supplemented with 7.5% fetal bovine serum (FBS). In vitro cell migration assay was performed using a BD Falcon cell culture insert (BD Biosciences). We seeded 1 × 10^5^ cells into the upper part of each chamber. Cells on the reverse side of the membrane were stained with 0.1% crystal violet. The migrating cells were counted under a microscope at 100 X at 6, 18, 24, 32 and 36 h.

We also conducted in vivo experiments, for which C57BL/6 mice were purchased from BioLASCO Co., Ltd. (Taiwan). When the mice were 6 to 8 wk of age, they were injected subcutaneously with 1 × 10^5^ LLC cells in the right flank. Our animal study was approved under the guidelines established by the Taipei Medical University Ethical Committee for Laboratory Animals. Tumor weights were documented on Days 15, 25, 40, and 47, or on the day of death.

3. The diffusion coefficient (D) was inversely related to the viscosity (*η*) of the microenvironment, in accordance with the Stokes-Einstein equation $D=\frac{k_{B}T}{6\pi \eta r}$ (where *T* is absolute temperature; *η* is viscosity; *r* is the radius of the particle; and *k*_*B*_ is the Boltzmann constant). Thus, the extent of cancer cell movement depends on the viscosity of surrounding microenvironments. Fillers form a barrier to hold down cancer cells. Cancer cells must overcome these obstacles by creating the “path-generating” way.

For the in vitro and in vivo experiments on manipulation of the microenvironment properties, mice were randomly divided into 3 groups (n = 5 animals/group for each time point). The 3 groups received the following treatments: cells only; cells plus HA (Restylane Perlane®, Q-Med AB, Sweden); and cells plus polyacrylamide (PAM) (Aquamid®, CONTURA, Denmark). The dynamic viscosity of the LLC, LLC + HA and LLC + PAM cancer samples (0.5 gm) at different time points was measured using a Cone & Plate CAP-2000 Viscometer (Brookfield Engineering Labs., Inc., Middleboro, MA, USA) at 37 ± 0.1°C. The shear rate was 237.6 s^-1^. The unit of dynamic viscosity is the poise (P). Different fillers were injected around the tumor surface on the seventh day of injections.

## Results

### CAM expressions of benign muscle tumors decrease as RTOP simulation

**Figure 1:**
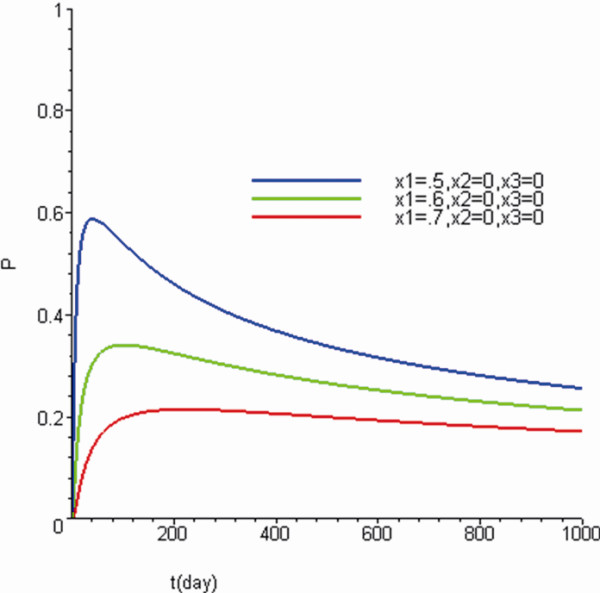
**The simulation indicated that RTOP (return to origin probability) gradually approached as low level as the tumor increased (*x***_**1**_ **= 0.5 to 0.7 mm) or as time passed (0 to 1000 days) (D = 10**^**-6**^**/*d*·*mm***^**2**^**).** The fractal factors were *α* = 0.4 and *β*_1,2,3_ = 0.9.

The simulation of Eq. (2) showed that RTOP (CAM concentration ratio $c\left(\vec{r},\hat{t}\right)$) gradually approached a low level as the tumor grew or with the passage of time under a tumor-specific fractal speed (Figure1).

**Figure 2:**
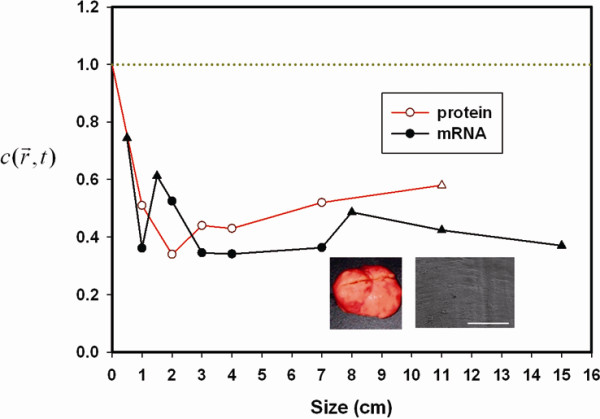
**Integrin*α***_***ν***_**mRNA and protein of uterine leiomyoma (n = 10), which were normalized to GAPDH and normal uterine tissues, showed a descending slope and approached a low constant level as the tumor size increased (mRNA = 0.39 ± 0.06 and protein = 0.47 ± 0.08).** The experiments were performed in duplicate. The surface topology of uterine leiomyomas revealed a relatively smoother contour correlated with their gross morphology than normal tissues (inset pictures).

The integrin *α*_*ν*_ of uterine leiomyoma (benign muscle tumor) was normalized to GAPDH and healthy tissues. The values for this parameter showed a descending slope and approached a low level as the tumor size increased (e.g., uterine leiomyoma: mRNA = 0.39 ± 0.06 and protein = 0.47 ± 0.08) (Figure2).

### The positional probability (CAM expression) of different cancer cells showed a biphasic pattern

**Figure 3:**
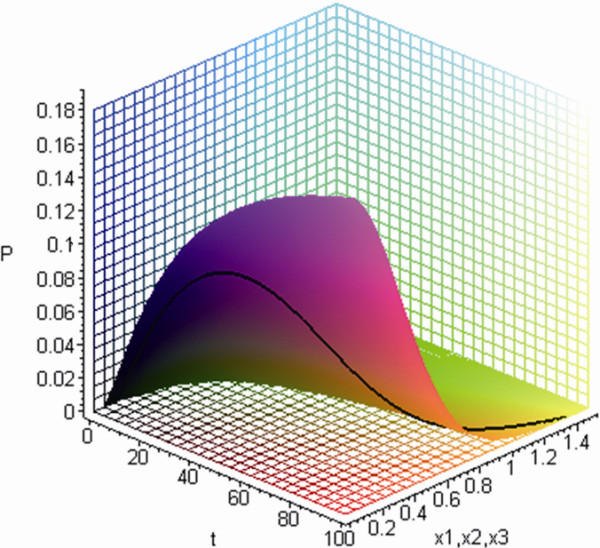
**In the simulation, positional probability showed a biphasic pattern with an initial increase followed by a decrease, for time and location (*x***_***1***_ ***= x***_***2***_ ***= x***_***3***_***, D***_***11***_ ***= D***_***22***_ ***= D***_***33***_ ***= 0.01/d·mm***^***2***^**,*α*** **= 0.4 and*β***_**1,2,3**_ **= 0.9).** The solid line indicates the curve under a specific *x*_*1*_ *= x*_*2*_ *= x*_*3*_ = 0.5 location value.

**Figure 4:**
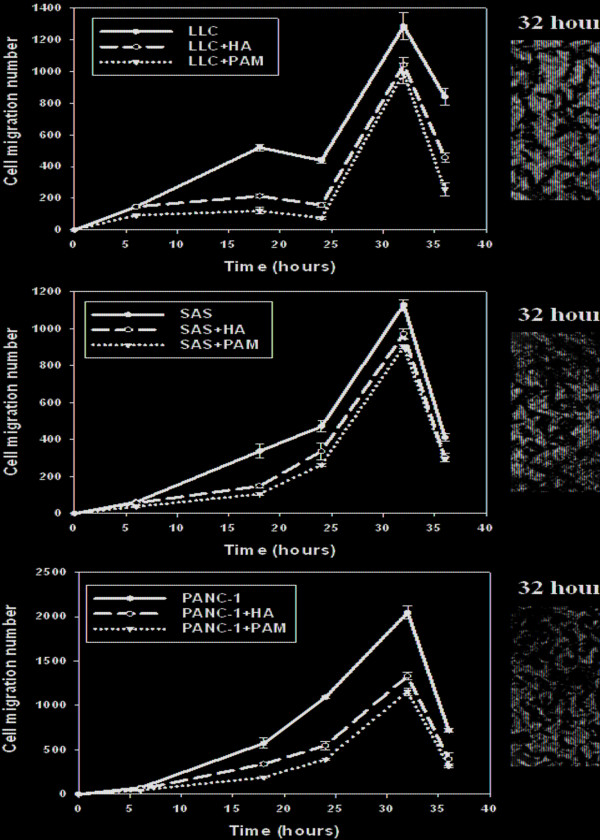
**The numbers of LLC (Lewis Lung Cancer), human squamous cell carcinoma (SAS), and human pancreatic carcinoma-1 (PANC-1) cells penetrating the membrane at specific locations represented the biphasic pattern in the cell migration assay.** The pictures show LLC, SAS and PANC-1 cells at 32 hours in each corresponding figure. All data represent the means **±** standard error (S.E.) from duplicate experiments (n = 3 for each time point).

**Figure 5:**
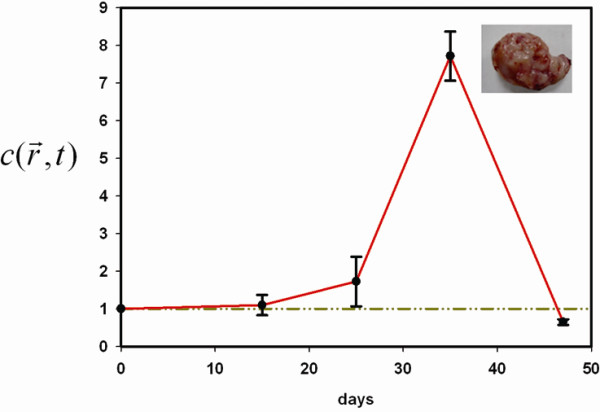
**Integrin*α***_***ν***_**concentration ratio also revealed a biphasic pattern associated with tumor growth in C57BL/6 mice.** Duplicate experiments were performed (n = 3 for each time point). The inset picture shows an LLC tumor.

The simulated curve of positional probability and molecular expressions in Eq. (1) revealed a biphasic pattern of an initial increase followed by later decline, also known as Lévy flight (Figure3). In vitro cell migration assay revealed that the number of three different stained cell lines lodged at specific locations underneath the membranes displayed a biphasic pattern, regardless of the type of filler added to the microenvironment (Figure4). In vivo experiment with C57BL/6 mice subcutaneously injected with LLC, integrin *α*_*ν*_ expressions also confirmed this trend (Figure5). 

### Manipulating microenvironment plasticity altered cancer behavior

**Figure 6:**
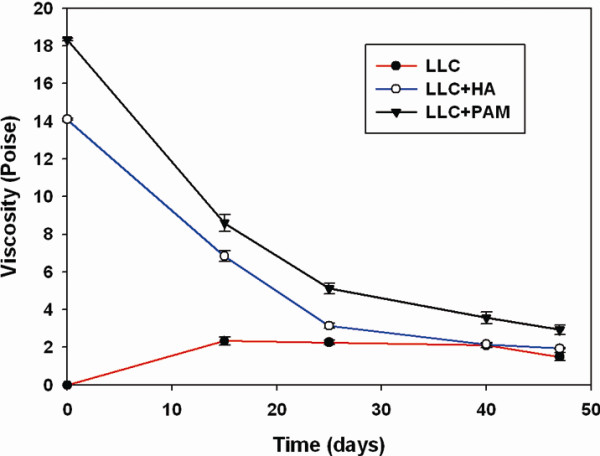
**Different fillers added to the microenvironments yielded different values for dynamic viscosity (LLC + HAs (hyaluronans) dynamic viscosity = 14.1 Poise) < < LLC + PAMs (polyacrylamides) dynamic viscosity = 18.3 Poise)) (*p*** **< 0.05).** Duplicate microenvironment plasticity experiments were performed (n = 3 for each filler group).

**Figure 7:**
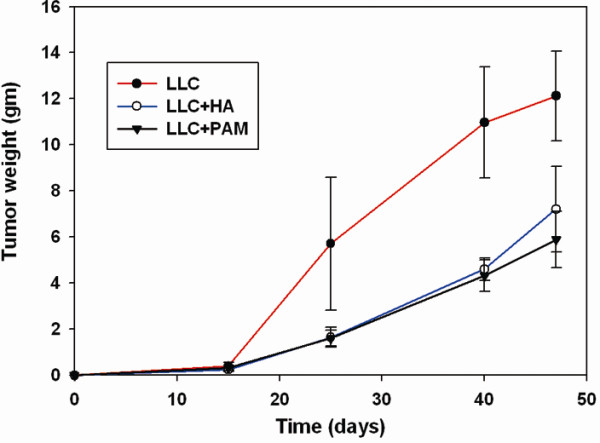
**Fillers with higher viscosity decreased the LLC tumor weight significantly (*p*** **< 0.05).** All data represent the means ± S.E. of duplicate experiments (n = 3 for each time point).

The experiments on microenvironment plasticity, together with Eq. (1), showed that cancer cell migration depended on heterogeneity or on the diffusion coefficients of microenvironments. The Stokes-Einstein equation indicates that higher viscosity (heterogeneity) of materials reduces the diffusion coefficient (*p* < 0.01, Figure6). In vivo experiments, the fillers caused tumor weight to decrease markedly (*p* < 0.01, Figure7).

#### Statistical analyses

Statistical results are presented as the mean ± standard error (S.E.). To compare the data from individual cancer groups, differences in tumor weight were tested by the Welch test (overall comparison) and post hoc Dunnett test (multiple comparisons). Differences in dynamic viscosity among the filler groups were assessed using the non-parametric Kruskal-Wallis H test and post hoc Dunnett test. All tests were 2-tailed and differences were considered significant if *p* < 0.01.

## Discussion

Tumorigenesis involves multiple and complex network mechanisms. To date, single targeted molecules have not yielded the expected rewards for diagnosis and treatment. Our modeling strategy simulated the so-called drunken unstable cancer cell migration as a fractal diffusion or random walk. Fractal factors can quantify the heterogeneity and anisotropy of tumors and their microenvironments. The positional probability can easily be normalized to a surface molecule expression (e.g., CAMs) of cancer cells as a cell-CAM-ECM module because cells and microenvironments reshape and interact with each other. Three possibilities describe the location of cancer cells, namely (1) stay in situ and coalesce to a mass; (2) move but return to origin; and (3) migrate to neighboring or distant sites. Briefly, we posit a new cancer anomalous diffusion hypothesis, as follows: motility dynamics underlie a cell-CAM-ECM module and relevant malignant/benign behaviors.

Researchers have long understood that dynamical reciprocity (DR) creates various dynamic space-time patterns. The monitoring of CAM expressions seems to provide net biological information without myriad complex intracellular signaling detections or targeted treatments. If no cancer cell exists or lodges in a specific space-time, no related CAM is generated to adhere to ECM. This phenomenon is accepted in anoikis (integrin-mediated death and caspase-dependent apoptosis) [25]. Namely, a specific CAM level corresponds to a unique cell-CAM-ECM interface (module).

Our model simplifies the construction and obtains profound results based on these assumptions. First, the concept of RTOP has been well applied to explain why the filaments in the molecular motor ratchet can always return to their original position after contraction or action in one or two dimensions [26]. In our study, RTOP is applied to explain the probability of benign or malignant cells which stay, seed or return to the original position or solid mass state (tumor self-seeding). Benign tumors always hold their solid tumor shape; but the shape or margin of malignant tumors (cancers) blur. Several benign tumors have self-limiting invasion features, including uterine leiomyoma, breast fibroadenoma, benign prostate hypertrophy, and brain meningioma. Namely, CAM expressions in the benign tumors steadily follow our RTOP prediction compared to the malignant tumors. The simulation revealed that CAM concentration ratio decreased as a benign tumor enlarged in the random structures of the microenvironments. If we selected a specific microenvironments (the periodic lattice) from all possible biological microenvironments, the data of benign tumors (uterine leiomyoma) growing in the periodic muscle structures approached the 3D Pólya random walk constant (0.340537). The RTOP value may be clinically significant to the treatment goals of molecular target inhibition. Namely, the tumor state may be suppressed to a state as characterized by the CAM expressions with little spatiotemporal variance in a regular-structure microenvironment.

Second, the biphasic pattern of the positional probability indicates the main process of cancer progression, namely initial growth with later spread, with a continued parallel cancer progression. Cancer cells are initially located in certain vicinity with a peak at a specific space-time; subsequently, most cancer cells begin to escape. Non-linearity violates chronological sequences of events; however, the main pattern remains evident. CAM expressions of cancers may help indicate the state and pattern of cancer in the clinical implications.

Third, the Stokes-Einstein equation provides an evaluation guide to microenvironment plasticity or the use of applied fillers in cancer control. Artificial control of microenvironments, including boundary conditions in differential equations, can impede the spread or migration of cancer cells. Our experiments showed that higher viscosity of fillers was associated with slower cancer migration or tumor growth. Manipulating ECM components provides information on how cancer progression might be attenuated. The results of our modeling experiments, especially those for RTOP and the use of fillers, indicate that our mathematical model may be useful in the development of new strategies for cancer prevention and treatment.

## Conclusions

We propose that complex diseases require dynamic, modular assessment and control. Biological networks appear to exhibit modularity as topological structures. The interplay between the positional probability and cell-CAM-ECM module shown in our study led us to establish a simplified mathematical model, and further provides useful information on cancer progression.

## Abbreviations

CAM, Cell adhesion molecule; DR, Dynamic reciprocity; ECM, Extra-cellular matrix; EMT, Epithelial to mesenchymal transition; HA, Hyaluronan; LLC, Lewis lung cancer; LMC, Lung metastatic colony; MET, Mesenchymal to epithelial transition; PAM, Polyacrylamide; PANC-1, Human pancreatic carcinoma-1; RTOP, Return to origin probability; SAS, Human squamous cell carcinoma; SEM, Surface electron microscopy; 3D, Three-dimension; TNM, Tumor-Node-Metastasis.

## Competing interest

The authors declare that they do not have any competing interests.

## Authors’ contributions

FCT created the modeling; FCT and MCW developed the equations; and FCT wrote the manuscript. JFL, CMC and FCT designed and performed the experiments. All the authors have read and approved the final manuscript.
